# SLC6A4 Gene Methylation in Premature Infants Undergoing Kangaroo Mother Care: A Prospective Longitudinal Study

**DOI:** 10.3390/biomedicines14061269

**Published:** 2026-06-02

**Authors:** Bruna Suzarte Campelo, Maria Clara de Magalhães-Barbosa, Aline de Araújo Brasil, Patrícia de Padua Andrade Campanha, Leo Travassos Vieira Milone, Paulo Victor Barbosa Eleuterio dos Santos, Stephanie Cristina Alves de Oliveira Saide, Vitor Barreto Paravidino, Jaqueline Rodrigues Robaina, Mariana Barros Genuino de Oliveira, Antonio José Ledo Alves da Cunha, Arnaldo Prata-Barbosa

**Affiliations:** 1 Department of Pediatrics, D’Or Institute for Research and Education (IDOR), Rio de Janeiro 22281-100, RJ, Brazil; brusuzarte@ufrj.br (B.S.C.); mariaclara.magalhaes@idor.org (M.C.d.M.-B.); aline.brasil@idor.org (A.d.A.B.); leo.milone@idor.org (L.T.V.M.); paulo.eleuterio@idor.org (P.V.B.E.d.S.); stephanie.saide@idor.org (S.C.A.d.O.S.); jaqueline.robaina@idor.org (J.R.R.); mariana.genuino@idor.org (M.B.G.d.O.); acunha@medicina.ufrj.br (A.J.L.A.d.C.); 2 Department of Neonatology, Leila Diniz Maternity Hospital, Lourenço Jorge Municipal Hospital, Rio de Janeiro 22793-000, RJ, Brazil; patcampanha@gmail.com; 3 Department of Pediatrics, School of Medicine, Federal University of Rio de Janeiro (UFRJ), Rio de Janeiro 21941-971, RJ, Brazil; 4 Department of Epidemiology, Institute of Social Medicine, State University of Rio de Janeiro (UERJ), Rio de Janeiro 20550-900, RJ, Brazil; vparavidino@gmail.com; 5 National School of Public Health (ENSP), Oswaldo Cruz Foundation (FIOCRUZ), Rio de Janeiro 21041-210, RJ, Brazil; 6 Department of Physical Education and Sports, Naval Academy, Brazilian Navy, Rio de Janeiro 21012-350, RJ, Brazil

**Keywords:** DNA methylation, *SLC6A4*, serotonin transporter, Kangaroo Mother Care, preterm infants, epigenetics, neonatal intensive care

## Abstract

**Background/Objectives:** Kangaroo Mother Care (KMC) has been proposed as a protective intervention that may modulate the epigenetic regulation of stress-related genes, such as *SLC6A4*, which encodes the serotonin transporter. Few studies have explored this association in humans. This study aimed to evaluate whether KMC affects the methylation status of *SLC6A4* in preterm newborns. **Methods:** This longitudinal observational study included preterm infants with birth weight ≤ 1800 g and gestational age between 25 and 34 weeks. Blood samples were collected at birth, Neonatal Intensive Care Unit discharge, and hospital discharge. Methylation levels at 13 CpG sites within the *SLC6A4* promoter region were quantified by bisulfite conversion and pyrosequencing. Methylation dynamics were analyzed using linear mixed-effects models adjusted for clinical covariates. **Results:** 75 preterm infants were analyzed (51 KMC; 24 non-KMC). Methylation levels ranged from 0.78% to 10.76% across all CpG sites and remained stable over time. At hospital discharge, the KMC group exhibited lower methylation at CpG6 than the non-KMC group (median = 0.96% vs. 1.21%, *p* = 0.021), but this difference was not statistically significant after correction for multiple testing. No significant differences were observed at other sites or in longitudinal methylation trajectories between groups. **Conclusions:** KMC was not associated with major longitudinal changes in *SLC6A4* methylation during the neonatal period. The nominal difference at CpG6 should be interpreted as exploratory and warrants further investigation. Larger, multicenter studies with long-term follow-up are needed to clarify the epigenetic mechanisms linking early caregiving experiences with stress regulation and neurodevelopmental outcomes in preterm infants.

## 1. Introduction

Prematurity is associated with developmental changes throughout life, and this risk is inversely proportional to gestational age. The pathophysiology of these changes is multifactorial. Epigenetic changes associated with adverse experiences in the Neonatal Intensive Care Unit (NICU) can alter the activity of stress-related genes, such as *SLC6A4*, which encodes the serotonin transporter, leading to developmental disorders and behavioral changes observed in premature infants [1].

**Figure 1:**
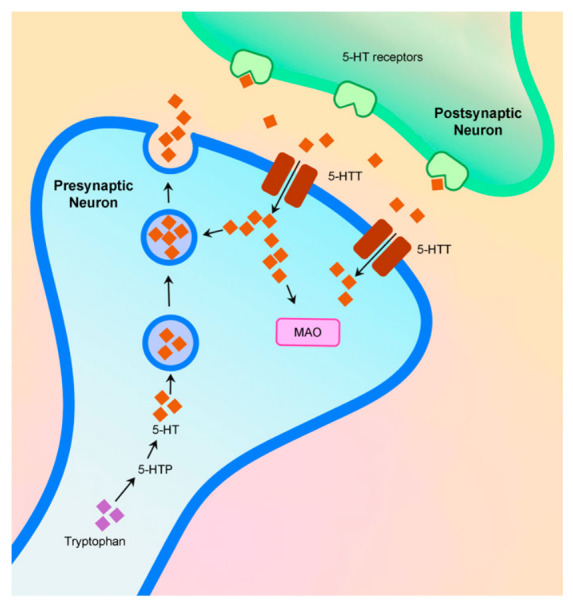
Schematic of the serotonergic synapse. The serotonin transporter (5-HTT), encoded by the *SLC6A4* gene, is the primary mechanism for serotonin (5-HT) reuptake from the synaptic cleft, regulating the duration and intensity of the signal, and is the focus of this study.

Serotonin is a neurotransmitter that influences a range of developmental outcomes, including behavior, cognition, emotion, and stress regulation. Serotonin receptors are located both in the central nervous system and in peripheral tissues. When serotonin binds to a receptor, it activates a cascade of events within the neuron, which can be excitatory or inhibitory, resulting in a cellular response [2,3]. A crucial component of the serotonergic system is the serotonin transporter, which removes serotonin from the synapse cleft. By reuptake of serotonin, the transporter controls the duration and intensity of serotonergic signaling. The faster serotonin is reuptaken, the shorter its duration in the cleft (Figure 1). The serotonin transporter is produced by the *SLC6A4* gene, which is highly expressed in the central nervous system. Several mechanisms regulate *SLC6A4* expression, including genetic variations. Many studies suggest that the promoter region of the serotonin transporter is associated with polymorphisms that influence personality traits, behavior, and susceptibility to socioemotional stress [4,5,6,7,8]. This region can exhibit short or long allelic variants, with the short variant linked to reduced serotonin transporter transcription and a high risk of adverse developmental outcomes, such as emotional and affective disorders in adolescence and adulthood [4,9]. Beyond genetic variation, the promoter region of *SLC6A4* is also subject to epigenetic regulation via DNA methylation, which may represent an additional layer of control that can be modulated by early environmental exposures.

Based on animal studies and rare human studies, it is speculated that interventions and protective care in the NICU may influence epigenetic alterations in some genes [10,11,12,13,14,15]. In genes related to stress and neurodevelopment, such as *SLC6A4*, these mechanisms have been little investigated in humans [12,14,15]. The Kangaroo Mother Care method (KMC) recommended in Brazilian neonatal units is a model of qualified and humanized care for newborns and their families [16]. The KMC may serve as a protective intervention, contributing to the reversal or prevention of certain epigenetic alterations, leading to modified outcomes in this population. A systematic search of PubMed and Lilacs using the terms ‘Kangaroo Mother Care’, ‘skin-to-skin contact’, ‘*SLC6A4*’, and ‘methylation’ did not retrieve any human studies that specifically examined this association. The present study aims to describe methylation of the *SLC6A4* gene in a cohort of low-birth-weight preterm infants and to compare results between groups that received and did not receive KMC.

## 2. Materials and Methods

### 2.1. Study Design and Study Population, and Ethics

This is an observational, longitudinal, prospective study that evaluated a cohort of premature newborns with birth weights ≤ 1800 g and gestational ages between 25 and 34 weeks, consecutively admitted to the NICU of a Brazilian public maternity hospital, from March 2020 to March 2021. Patients with major congenital malformations, clinical suspicion, or laboratory confirmation of genetic syndromes were excluded from the study. The maternity hospital where the study was conducted, Maternidade Leila Diniz, is located at a municipal hospital in the city of Rio de Janeiro, Brazil, and a state and national reference center for the humanized care of low-birth-weight newborns, with a particular focus on Kangaroo Mother Care.

### 2.2. The Kangaroo Mother Care Method

In Brazil, the recommended KMC combines biopsychosocial intervention strategies with an environment that favors the care of low-birth-weight premature infants and their families, promoting parental and family participation in neonatal care. The method includes skin-to-skin contact, which begins early and progresses from touch to the kangaroo position. The kangaroo position involves placing the newborn, wearing only diapers, in an upright position next to the parents’ chests for the minimum time necessary to stabilize the baby and for as long as both parties consider pleasurable and sufficient. It should be performed in a safe, guided manner, with care and support from an appropriately trained healthcare team [16]. The first stage of the KMC is performed in the NICU, where intermittent skin-to-skin contact begins, but the mother does not remain hospitalized. The second stage takes place in the neonatal intermediate care unit, where the mother remains hospitalized with the newborn full-time. The third stage occurs at home following hospital discharge and involves maintaining skin-to-skin contact and monitoring the newborn until the baby reaches 2500 g [16].

As a national reference center for KMC since 1999, this maternity ward routinely applies the method, and no specific implementation process was conducted for this study. All preterm infants who fully participated in the first two (in-hospital) stages of the KMC were included in the KMC group; preterm infants who did not participate in any stage, either by family choice or because their parents were unable to remain in the maternity ward, constituted the non-KMC group. Patients excluded after entry into the study due to death or transfer from the NICU, as well as patients who did not fully participate in the two in-hospital stages of the KMC, were considered losses.

### 2.3. Ethics

This study was approved by the research ethics committees of the participating institutions (IDOR-RJ-2020 # 4.208.064, SMS-RJ-2020 # 4.353.102), and written informed consent was obtained from all parents or legal guardians of the newborns. All data were anonymized.

### 2.4. Data Collection and Clinical Variables

Blood samples for *SLC6A4* gene methylation analysis were collected at three time points: from cord blood at birth (D1); from peripheral blood on the day of NICU discharge (D2); and on the day of hospital discharge (D3). All samples were collected by a NICU neonatologist in EDTA tubes, stored at 2–8 °C in the maternity ward for up to 72 h, a condition previously shown to preserve relative stability of global DNA methylation profiles [17]. They were then transported to the epigenetics laboratory, where they were aliquoted and stored at −80 °C until processing.

Sociodemographic and clinical data were extracted from patient records. Maternal and neonatal variables related to pregnancy, delivery, and NICU stay were collected, including diagnoses and treatments received, clinical outcomes, and *SLC6A4* gene methylation results. Data were entered into REDCap (Vanderbilt University, Nashville, TN, USA) and later exported to Excel spreadsheets (Microsoft Brazil, São Paulo, Brazil).

### 2.5. Procedures for Gene Methylation Analysis

**Figure 2:**
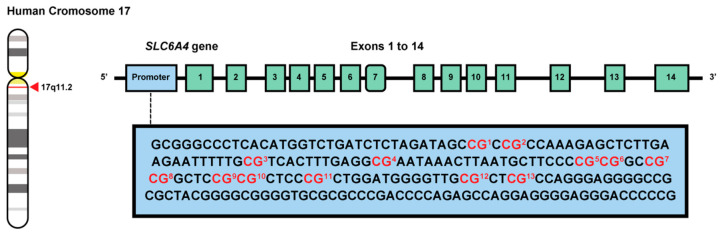
Diagram of the *SLC6A4* gene on chromosome 17 (17q11.2), demonstrating the sequence of 13 CpG sites in its promoter region.

The region of the *SLC6A4* gene analyzed consisted of the CpG islands in the promoter region (chr17:28562750-28562958, Human hg19 Assembly), spanning from −69 to −213, corresponding to 13 CpG sites adjacent to exon 1 (Figure 2). Genomic DNA was extracted using the DNeasy Blood & Tissue Kit (Qiagen, Hilden, Germany). DNA concentration and purity were measured spectrophotometrically at 260, 280, and 230 nm using a NanoDrop 2000c (Thermo Fisher Scientific, Waltham, MA, USA). Integrity was assessed by 1% agarose gel electrophoresis.

Sodium bisulfite conversion was performed using the EZ DNA Methylation kit (#D5002, Zymo Research, Irvine, CA, USA). After DNA conversion, samples were amplified using the PyroMark PCR kit (#978703, Qiagen). Amplifications were performed in a final reaction volume of 50 μL, containing 0.2 μM of each primer, 2.0 μL of converted DNA, and the Master Mix provided in the kit. A ProFlex™ 3 x 32-well PCR System (Thermo Fisher Scientific, Waltham, MA, USA) or a Veriti 96-Well thermal cycler (Applied Biosystems, Waltham, MA, USA) was used. All amplification reaction products were analyzed by 1% agarose gel electrophoresis to verify the correct amplification size in the L-PIX gel photodocumentation system (Loccus Biotecnologia, São Paulo, Brazil). Therefore, later, only one of the amplified strands could be selected, so one of the oligonucleotides used in the PCR reaction was labeled with biotin.

Biotinylated PCR products in a total volume of 10 μL were immobilized on 3.0 μL of magnetic streptavidin-coated Sepharose beads (PyroMark Q48 Magnetic Beads, Qiagen, Hilden, Germany). Pyrosequencing was then performed using PyroMark Q48 Advanced CpG reagents on the PyroMark Q48 Autoprep system (both from Qiagen). The methylation percentage of each CpG site was automatically generated by the PyroMark Q48 Autoprep software (v. 2.4.2) with default quality control settings. Primer design for PCR and pyrosequencing were performed using PyroMark Assay Design 2.0 software (Qiagen). The commercial human methylated and non-methylated DNA set (Human Methylated & Non-methylated DNA Set, Zymo Research, CA, USA) was used as a positive and negative control in the methylation detection assays. All PCR and pyrosequencing primers, as well as the sequences analyzed, are detailed in Table S1 (Supplementary Material).

### 2.6. Statistical Analysis

KMC was categorized as a binary variable (KMC vs. non-KMC), because no associations were observed between length of stay in KMC and total methylation or methylation at specific sites, measured at NICU discharge and hospital discharge (Supplementary Material, Table S2).

Comparisons of demographic and clinical variables between the KMC group and the non-KMC group were made using appropriate statistical tests, depending on the desired clinical interpretation and the behavior of the variables: the chi-square test or Fisher’s exact test for proportions and the Mann–Whitney test for medians.

Methylation percentages were expressed as mean and standard deviation, median, and interquartile range. The medians of the two groups were compared using the Mann–Whitney U test, and the medians of the three time points were compared using the Kruskal–Wallis test.

Longitudinal analyses of methylation percentage over time were performed for each CpG site and the average of the 13 CpG sites combined (total methylation) using linear mixed-effects models. Models were fitted to our data, including the variables time, group, time*group interaction, and sociodemographic and care-intensity variables, which could influence epigenetic marks.

**Figure 3:**
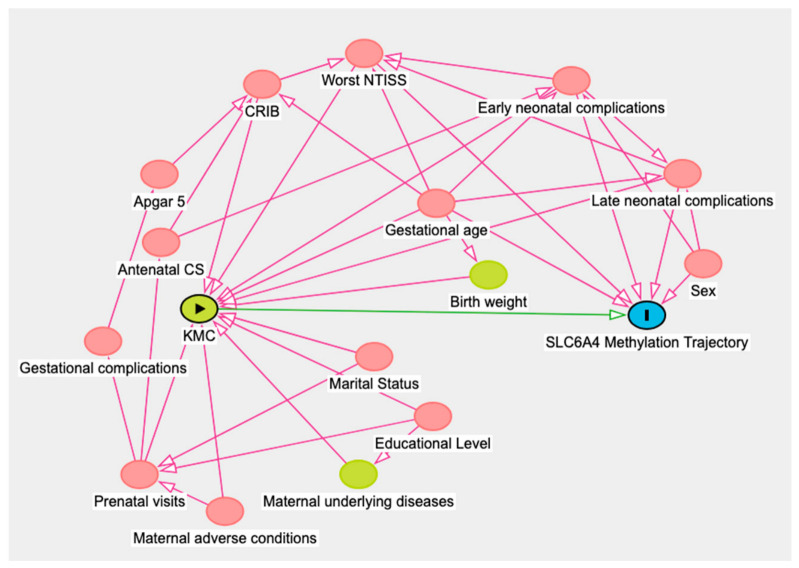
Directed acyclic graph (DAG) representing the causal paths between Kangaroo Mother Care (KMC) and *SLC6A4* methylation trajectory. The nodes are color-coded according to their role in the causal model: the green node with a triangle represents the exposure (KMC); the blue node represents the outcome (*SLC6A4* methylation trajectory); red nodes represent ancestors of both the exposure and the outcome (potential confounders); and green nodes represent ancestors of the outcome only (competing exposures or predictors of the outcome). **Maternal underlying diseases:** chronic hypertension, hypothyroidism, psychiatric disorders, diabetes, asthma, obesity. **Maternal adverse conditions:** childhood violence, domestic violence, illicit drugs, smoking, alcoholism, homelessness. **Early neonatal complications:** respiratory distress syndrome, pulmonary hemorrhage, early sepsis, patent ductus arteriosus. **Late neonatal complications:** intracranial hemorrhage, periventricular leukomalacia, late sepsis, bronchopulmonary dysplasia, necrotizing enterocolitis, retinopathy of prematurity.

To identify potential confounding variables for the causal association between Kangaroo Mother Care and *SLC6A4* methylation changes over time, we developed a Directed Acyclic Graph (DAG). The DAG framework incorporated maternal and gestational factors (education, marital status, underlying diseases, gestational complications, antenatal corticosteroids, adverse conditions and prenatal care) as well as neonatal variables (gestational age, birth weight, sex, Apgar at 5 min, CRIB (Clinical Risk Index for Babies) and the highest NTISS (Neonatal Therapeutic Intervention Scoring System), early and late neonatal complications) (Figure 3).

Among the four minimal sufficient adjustment sets identified by the DAG, we selected the following set for inclusion in our multivariable models: sex, gestational age, early neonatal complications, late neonatal complications, and the highest NTISS score. This approach ensures that the causal effect is estimated while avoiding overadjustment and bias.

Autoregressive, exchangeable, independent, and unstructured covariance matrix structures were tested in the models, and the appropriate structure was selected for each site based on the quasi-likelihood parameter under the independence model criterion. The trend in methylation percentage over time (increase or decrease) was assessed within each group and compared between groups.

Effect size was estimated using the partial Cohen d calculation, dividing the beta coefficient of the difference between the KMC and non-KMC slopes (adjusted difference between changes over time) by the model’s residual standard deviation. The thresholds for Cohen’s D effect sizes were 0.20 (small), 0.50 (medium), and 0.80 (large) [18].

In assessing methylation differences between the two groups at each of the 13 CpG sites, the Benjamini–Hochberg method was used to correct for multiple testing when nominal differences were statistically significant. A formal a priori power calculation was not performed due to the lack of pilot data on KMC-specific *SLC6A4* methylation. Thus, because of the exploratory nature of this study, with a limited sample size and unbalanced groups, a false discovery rate [FDR] of 10% (q < 0.10) was allowed to balance the risk of Type I errors (false positives) against the risk of Type II errors (missing potentially meaningful biological signals in a vulnerable, hard-to-reach population). This threshold implies that among the results identified as significant, 10% are expected to be false positives. This level is often considered acceptable in discovery-phase studies where the goal is to identify candidates for further validation rather than to provide definitive evidence of association.

The statistical programs used were R version 4.5.3 (R Core Team, Vienna, Austria) and SAS On Demand for Academics version 9.4 (SAS Institute, Inc., Cary, NC, USA).

## 3. Results

### 3.1. Characteristics of the Study Population

**Figure 4:**
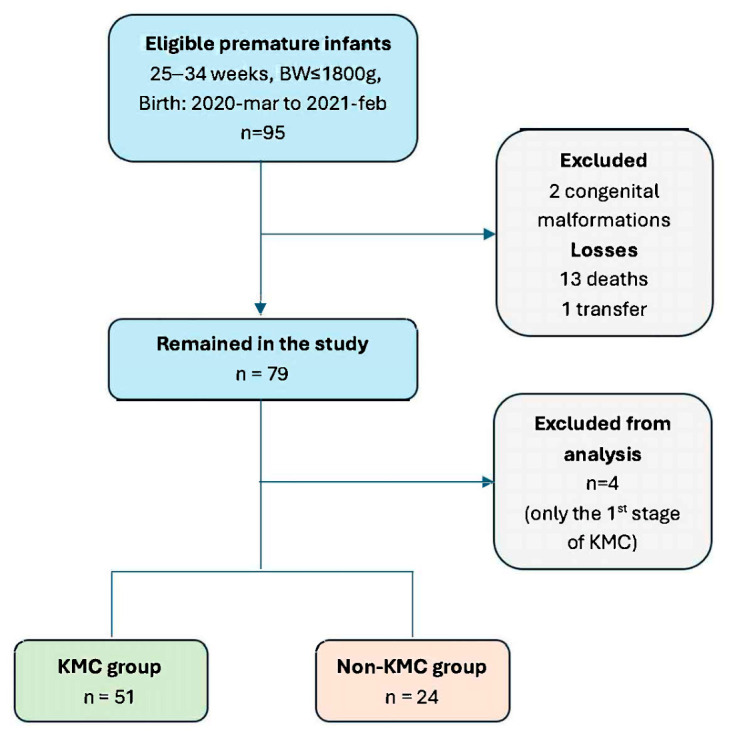
Flowchart of participant inclusion in the study. BW—Birth weight; KMC—Kangaroo Mother Care method.

Of the 95 eligible preterm infants, two had congenital malformations and were excluded, thirteen died, and one was transferred during their NICU stay. Of the remaining 79, four who initiated the first stage of KMC did not continue and were excluded from the analyses. Therefore, 51 preterm infants participated in both in-hospital stages of Kangaroo Mother Care (KMC group), and 24 did not participate in any stage (non-KMC group) (Figure 4).

Regarding maternal characteristics, there were no significant differences between the two groups regarding maternal age, race, parity, underlying diseases (including chronic hypertension, hypothyroidism, psychiatric disorders, diabetes, asthma, and obesity), adverse health conditions (including childhood violence, domestic violence, illicit drug use, smoking, alcoholism, homelessness), antenatal corticosteroid use, or type of delivery. However, mothers in the KMC group had a higher level of education (*p* = 0.015), a higher proportion were married or in a stable relationship (*p* = 0.002). They had attended more than six prenatal care visits (*p* = 0.011) (Table 1).

Regarding neonatal characteristics, there were no significant differences between the groups in sex, birth weight, gestational age (GA), weight-to-GA ratio, history of resuscitation in the delivery room, or 5 min Apgar score. Twins were only observed in the KMC Group (15.7%) (Table 1). There were also no significant differences in severity scores (CRIB, SNAPE II, and NTISS) or most morbidities, except for the use of parenteral nutrition, which was less frequent among preterm infants in the KMC Group (56% vs. 83.3%, *p* = 0.047). The length of stay in the neonatal ICU and the total length of hospital stay were also shorter in the KMC Group (medians of 9 days vs. 13.5 days and 29 days vs. 43 days, *p* = 0.01, respectively) (Table 2).

### 3.2. KMC Implementation Results

Kangaroo Mother Care (KMC) was primarily performed by mothers in both stages, with occasional participation by fathers or other caregivers during stage 1. The results regarding the method revealed an early start to the intervention, with a median age of 3 days (IQR: 2–5.5). The first stage had an average duration of 19.2 ± 16.8 days, with a daily dedication of 2.3 ± 1.3 h. In the second stage, an increase in practice intensity was observed, with average daily hours rising to 5.3 ± 1.5 (median 6 h), and the average length of stay in this phase was 15.4 ± 7.2 days. Adding the stages together, the total length of stay of premature newborns in the method during hospital admission was, on average, 34.7 ± 20.1 days (median of 29 days) (Table 3).

### 3.3. SLC6A4 Methylation Analysis

**Figure 5:**
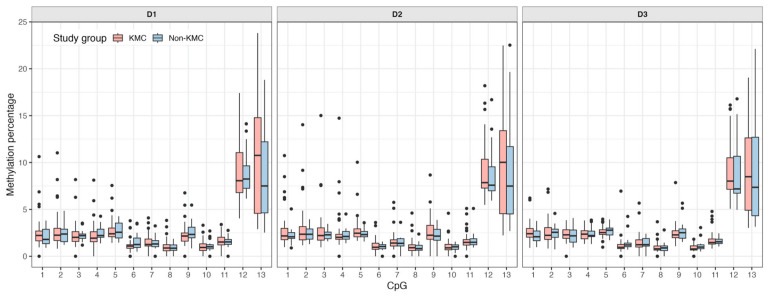
Methylation at CpG sites in the promoter region of the *SLC6A4* gene in the Kangaroo and Non-Kangaroo groups at three time points: at birth (D1), on the day of discharge from the Neonatal ICU (D2), and on the day of hospital discharge (D3). At hospital discharge (D3), a statistically significant difference was observed at CpG 6, with higher median methylation in the Non-Kangaroo group (1.21 [1.02–1.41] vs. 0.96 [0.82–1.26], *p* = 0.021).

Descriptive statistics are available in the Supplementary Material (Table S3). Median methylation percentages across all time points ranged from 0.78% to 2.78% for CpGs 1-11. Sites 12 and 13, showed consistently higher methylation levels, with medians ranging from 7.19% to 10.76%. No difference was observed in the total methylation of the *SLC6A4* gene between the KMC and non-KMC groups at any of the three time points. Regarding the specific CpG sites, no differences between groups were observed at any site at birth (D1) or at NICU discharge (D2). At hospital discharge (D3), a slight statistically significant difference was observed only at CpG 6, with higher median methylation in the non-KMC group (1.21 [1.02–1.41] vs. 0.96 [0.82–1.26]), but statistical significance disappeared after correction for multiple testing (Figure 5 and Supplementary Material [Table S3]).

**Figure 6:**
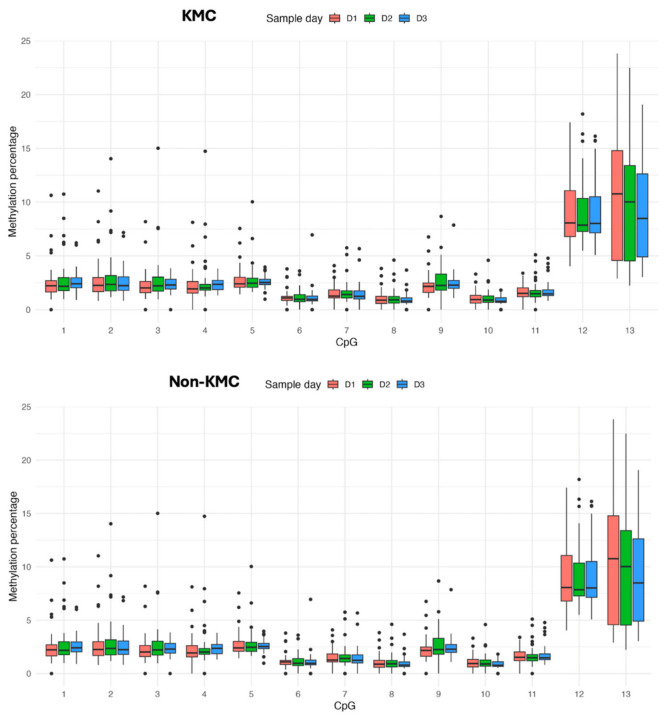
Comparing *SLC6A4* methylation between three time points in the Kangaroo Mother Care (KMC) and non-KMC groups. Methylation at 13 CpG sites in the promoter region of the *SLC6A4* gene at birth (D1), on the day of discharge from the Neonatal ICU (D2), and on the day of hospital discharge (D3) in the KMC and Non-KMC groups. There was no significant difference in methylation between the three days studied and when comparing two by two, from birth to discharge from the NICU (D1 and D2), from birth to discharge from the hospital (D1 and D3), and from discharge from the NICU to discharge from the hospital (D2 and D3).

There was no significant difference in methylations at each CpG site in both groups, between the three days studied, and in the two-by-two comparison between these days: birth and NICU discharge (D1 and D2), birth and hospital discharge (D1 and D3), and NICU discharge and hospital discharge (D2 and D3) (Figure 6).

There was also no difference in the total methylation of the *SLC6A4* gene between the KMC and non-KMC groups at any of the three time points (Table 4).

### 3.4. Longitudinal Methylation Trajectories

**Figure 7:**
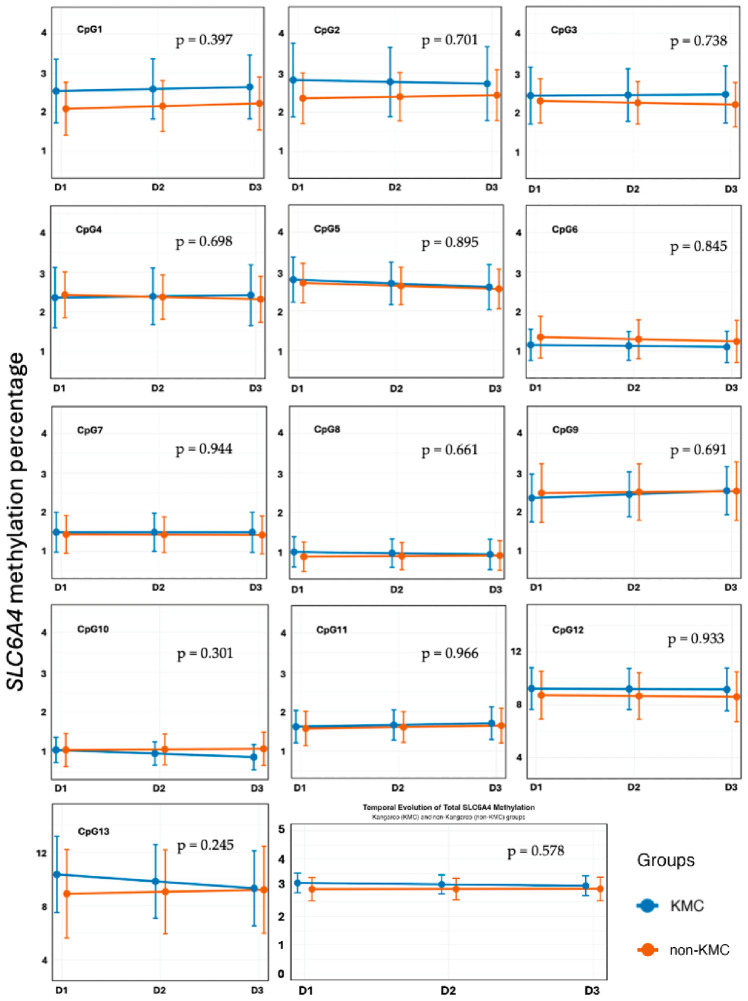
Temporal course of methylation in each group and comparison between groups, using mixed-effects models. There was no significant increase or decrease in the temporal evolution of each group, nor was there a significant difference between groups in the temporal evolution of methylation. *p*-values adjusted by the Benjamin-Hochberg method (False Discovery Rate—FDR).

Longitudinal analysis of the *SLC6A4* gene using linear mixed-effects models showed methylation remained stable over time in both groups (Table 5 and Figure 7).

In the KMC group, although eight CpGs exhibited negative beta coefficients and five CpGs showed positive coefficients, the values were close to zero and not statistically significant, indicating no consistent increase or decrease in methylation over time. The closest value to significance was at CpG 13 (β = −0.516; 95% CI: −1.1471 to 0.1158, *p* = 0.1077), suggesting a decreasing trend, but this was not statistically significant. In the non-KMC group, six CpGs showed negative beta coefficients. In comparison, seven CpGs showed positive coefficients near zero and were not statistically significant, indicating no consistent increase or decrease in methylation over time. The interaction term (difference between slopes) reflects the comparison between groups of the changes in methylation over time. The beta coefficients were close to zero in most cases, indicating that the trend lines of the two groups are virtually parallel and that no difference between the groups exists. Since no *p*-values reached statistical significance in the longitudinal mixed-effects models, no correction for multiple testing was applied to these analyses. CpG 13 showed the largest absolute difference between slopes (β = −0.6647), indicating that the KMC group tended to decrease methylation. In contrast, the control group tended to increase it slightly, but the high variability (reflected in the wide confidence interval: −1.79 to 0.46) prevented this difference from being significant (*p* = 0.245). Adjusted Cohen’s effect sizes were predominantly negligible (d < 0.10), with only CpG 13 reaching a small-to-moderate magnitude (d = 0.344).

## 4. Discussion

This study investigated the methylation profile and longitudinal trajectories at 13 CpG sites in the promoter region of the *SLC6A4* gene in preterm infants cared for in a reference maternity hospital for the Kangaroo Mother Care, comparing those who received KMC with those who did not. The results revealed low methylation percentages across most CpG sites analyzed and stability across the three collection points. The methylation percentages at sites 12 and 13 were higher than at other sites in both groups and across all three time points. While CpG6 showed a potential difference at hospital discharge, with lower methylation in the KMC group, and CpG 13 exhibited the large absolute difference in slopes, no consistent pattern of methylation changes was observed over time, either within or between groups, suggesting that KMC, within this specific hospital context and timeframe, was not associated with large-scale modifications in peripheral blood *SLC6A4* methylation in preterm infants during hospitalization. The negligible effect sizes reinforced the lack of clinical impact of the intervention on these specific epigenetic markers.

### 4.1. The Epigenetic Impact of Early Care

Human epigenetic research on prematurity is based on the premise that early experiences, both adverse and protective, can be “biologically embedded” through DNA methylation, thereby influencing neurodevelopment [19]. Animal studies have provided a foundation for this, demonstrating that the offspring of low-care mothers exhibit higher methylation and greater stress reactivity, which can be reversed by cross-fostering or pharmacological agents [10,20].

Compared with animal studies, obtaining scientific evidence in humans linking DNA methylation to stress in early neonatal life is more complex. The heterogeneity of the human population leads to several environmental and genetic confounding factors. Furthermore, because the brain is inaccessible for DNA methylation analysis in living humans, many studies are limited to peripheral tissues, such as saliva and white blood cells, whose relevance to brain pathophysiology remains uncertain. [21].

While the extent to which peripheral methylation reflects brain-specific regulation remains a subject of ongoing debate, the *SLC6A4* gene has been identified as a sensitive biomarker of stress and trauma across the lifespam [9]. Studies linked methylation of specific CpG sites in the promoter region of the *SLC6A4* gene to childhood adversities. The loss of a parent was associated with increased methylation at CpG2 [22]; childhood sexual abuse was associated with hypermethylation at CpGs 1 and 3 [23]; bullying was associated with increased methylation at CpG 8 in one of the twins of monozygotic pairs [24]. Along the same line, the study of epigenetic alterations in premature infants has focused on genes related to stress and neurodevelopment [6,25,26,27,28,29,30,31,32,33,34], and the *SLC6A4* gene has been a key focus of these studies [9,27,28,30,31,32,33,34,35].

### 4.2. Comparison with NICU Stress Research

Some studies in the same cohort of preterm infants have shown that exposure to pain-related stress and NICU stay can alter the activity of the serotonin transporter gene. A study analyzed 20 CpG sites in the promoter region of the *SLC6A4* gene in 56 very preterm newborns and 32 full-term newborns. The authors found no difference in methylation between the groups at birth. On the other hand, preterm infants exposed to high levels of pain showed increased methylation at CpG sites 5 and 6 between birth and discharge from the NICU. In contrast, methylation remained stable in preterm infants with low pain levels [27]. Another study also analyzed the methylation status at 20 CpGs of the *SLC6A4* promoter region in 48 very preterm and 30 full-term newborns. The authors observed no difference between the groups at birth. However, in preterm infants, methylation at CpG 5 was significantly higher at NICU discharge than at birth in full-term infants, and methylation at CpG 7 increased significantly between birth and NICU discharge [28]. These studies collectively suggest that prematurity alone is not associated with epigenetic alterations in the *SLC6A4* gene; however, exposure to pain-related stress and NICU stay may alter the activity of the serotonin transporter gene, indicating that epigenetic alterations result more from the type of NICU experience than from the length of stay.

In the present study, we did not quantify the pain level of preterm infants during their NICU stay, which prevents a direct correlation between stress and *SLC6A4* methylation. The subtle difference in methylation at CpG 6 at hospital discharge only in the non-KMC group was not robust after multiple testing correction. Although this finding should be interpreted solely as exploratory, the direction of the finding is consistent with the idea that KMC might act as a buffer against the hypermethylation typically induced by NICU stress. However, it is not possible to confirm whether a cumulative reduction in perceived stress mediated this. A study proposed that preterm infants are particularly sensitive to environmental modulation of stress response systems, with caregiving quality and maternal touch serving as potential buffers against adverse epigenetic programming [19]. On the other hand, contrary to our expectations, we found no difference between the KMC and Non-KMC groups in any other CpG. One possible, though speculative, explanation is that the institutional humanized care environment may have influenced the results. Since this institution is a national referral center for KMC, we hypothesize that even premature infants whose parents were unable to engage in the method for various reasons received more skin-to-skin contact and gentler handling from the healthcare team compared to conventional care. Although not directly measured, the degree of environmental contrast between the KMC and non-KMC groups may have been insufficient to produce measurable molecular-level changes.

Furthermore, in the present study, we did not compare *SLC6A4* methylation at birth between preterm infants and full-term newborns. However, in a recently published study [35], we made this comparison in a distinct cohort of newborns from another maternity hospital. We identified significant differences in *SLC6A4* methylation at birth in CpGs 12 and 13, with higher methylation in preterm infants. Other findings of that study were CpG 9 lower methylation in preterm infants at D5, the highest values of methylation in extremely preterm infants, and longitudinal mixed-effects analysis showing that total and site-specific methylation at CpGs 2, 8, and 9 increase over time in full-term infants, while methylation remained stable over time in very preterm and extremely preterm infants. These results are consistent with other studies on *SLC6A4* [32,33] and on genes related to stress and neurodevelopment, such as NR3C1 [25] and LINE-1 [26], which also found differences in the methylation patterns of preterm and full-term newborns at birth. These studies suggest that prematurity, per se, is an indicator of prenatal stress and is associated with a distinct methylation pattern at birth.

### 4.3. Temporal Stability of Methylation

The absence of significant longitudinal changes from birth to hospital discharge suggests that *SLC6A4* methylation in peripheral blood is relatively stable during the first weeks of life. This aligns with findings from Dokum et al. [29], who evaluated the total methylation of several genes related to neonatal stress, including *SLC6A4*, in 25 to 50% of fecal samples from 45 preterm infants aged 24–30 weeks, at two time points: between 7 and 14 days and at NICU discharge. The authors found no significant difference in the methylation of this gene between the two time points. According to Szyf (2019), while perinatal experiences can influence epigenetic regulation, such changes are often subtle and require either prolonged or repeated exposures to environmental stimuli to become detectable [21]. The low absolute methylation percentages observed in the present study (mostly below 3% at most CpGs) are consistent with prior reports in neonatal samples [27,29]. The higher levels at sites 12 and 13 were also observed in our other cohort, particularly in the extremely preterm infants [35]. These findings are intriguing and suggest that these specific CpGs might be under different regulatory control or represent a different baseline in early life. However, without functional data on gene expression, their specific biological impact remains speculative.

In addition, this study found no robust differences in longitudinal changes in *SLC6A4* gene methylation between those exposed and those not exposed to Kangaroo Mother Care. The predominantly negligible effect sizes (Cohen *d* < 0.10) observed across CpG sites are consistent with subtle epigenetic signals typically reported in the early adversity literature and reinforce the exploratory nature of the present findings. These results should be interpreted with caution. They may be attributed to the effect being nonexistent, the follow-up period being too short to detect epigenetic changes, or insufficient statistical power to detect subtle effects. On the other hand, the finding of lower methylation at CpG6 in the KMC group at discharge, and a difference methylation trends over time at CpG 13—though isolated and exploratory—warrants further investigation. We found no other studies on the effect of Kangaroo Mother Care on the *SLC6A4* gene methylation in preterm infants admitted to the NICU.

### 4.4. Other Studies on the Impact of Maternal Touch

While we found no studies assessing the impact of a stress-protective strategy, implemented continuously during the NICU stay, on *SLC6A4* gene methylation, some studies have evaluated the effect of maternal touch on stress-related genes after the neonatal period [12,13,14,15]. However, we found no studies assessing the impact of a stress-protective strategy, implemented continuously during the NICU stay on *SLC6A4* gene methylation. Wigley et al. performed a post hoc analysis of cohort data to evaluate the role of maternal touch during the stress response in 29 very preterm infants at 3 months’ corrected age, and its association with *SLC6A4* methylation, quantified at NICU discharge [15]. A low level of maternal touch during the assessment of emotional response to stress in preterm infants at three months intensified the effect of the association between hypermethylation at CpG2 and a high level of negative emotional response [15]. Conradt et al. demonstrated that adequate maternal touch and emotional support are associated with *NR3C1* methylation, another gene implicated in stressful situations, in five-month-old full-term infants [12]. Krol et al. observed reduced methylation of the oxytocin receptor gene (OXTR) between 5 and 18 months in full-term infants whose mothers were more engaged in caregiving and provided adequate maternal touch during the study period [13]. Fontana et al. reported that an early intervention based on maternal care and positive multisensory stimulation, initiated at 1 week of life, reversed birth hypomethylation of the LINE-1 promoter in 34 very preterm infants (25–29 weeks) to levels comparable to those of healthy term newborns at hospital discharge and improved neurodevelopmental outcomes at 12 and 36 months [14].

### 4.5. Long-Term Trajectories and Neurodevelopment

Chau et al. observed that exposure to pain-related stress in the neonatal intensive care unit associated with the COMT Met/Met genotype was associated with increased methylation at seven CpG sites in the *SLC6A4* gene, which in turn was related to behavioral problems in seven-year-old children who were born very preterm [34]. Mascheroni et al. observed greater emotional dysregulation in preschoolers who were born preterm compared to those born at term, and an association between negative emotional response to stress during childhood and increased methylation of the *SLC6A4* gene at 4.5 years of age [35]. This evidence suggests that stressful experiences in the neonatal intensive care unit (NICU) lead to altered methylation status of the gene encoding the serotonin transporter, with consequences for socioemotional development during childhood. A portion of the present study cohort was followed until 42 months of age to assess neurodevelopmental outcomes and to perform a new methylation analysis of the *SLC6A4* gene. The data are being analyzed for imminent publication.

### 4.6. Strengths and Limitations

This is the first study to specifically examine *SLC6A4* gene methylation in the context of KMC as a continuous stress protective strategy during the NICU stay. The longitudinal design, capturing three distinct time points, enabled a temporal trajectory analysis of *SLC6A4* methylation in very premature infants during a critical neonatal period. The use of advanced pyrosequencing and DAG-based confounding control adds significant methodological weight to the findings.

On the other hand, other methodological aspects may have limited our ability to detect epigenetic differences. The relatively small sample size (due to logistical and financial constraints), the low absolute methylation levels at most CpG sites, and the imbalance between groups (51 KMC vs. 24 non-KMC) likely limited the power to detect small effect sizes. It should be considered when interpreting null findings. However, this study was designed as an exploratory analysis of KMC’s effect on *SLC6A4*, which further justifies using a slightly higher FDR threshold to avoid discarding relevant trends. On the other hand, even with this more lenient threshold of 10%, no CpG site reached statistical significance after correction. This suggests that the observed nominal differences are either very small in magnitude or highly variable across individuals, reinforcing the need for caution to avoid false-positive interpretations. Thus, results need to be interpreted as exploratory and hypothesis-generating. While a formal a priori power calculation was not feasible due to the lack of prior data on the association between *SLC6A4* methylation and KMC, our sample size is comparable to that of other published epigenetic studies in neonatal intensive care.

Second, methylation in umbilical cord blood or in peripheral blood may not reflect gene regulation in brain tissues where *SLC6A4* is predominantly expressed. In addition to using samples from different biological materials, we did not account for heterogeneity in blood cell types, which can influence the methylation quantification. However, our main interest lay in comparing the temporal trends of methylation between groups rather than in precise quantification, which was possible with samples from the same patients collected over short periods.

Third, despite using Directed Acyclic Graph (DAG) modeling to control for potential confounders, residual confounding from unmeasured social or environmental variables cannot be ruled out. For example, it can be questioned whether the initial storage conditions for the samples (at 2–8 °C for up to 72 h) were adequate. Although some studies recommend shorter intervals between sample collection and processing for methylation analyses, this issue is still under debate. Furthermore, the methylation levels in our study remained stable over time, suggesting no systematic temporal drift or increased variability indicative of instability in methylation profiles associated with storage conditions. We believe that the pre-analytical conditions used did not introduce a relevant bias into the analyses. Another source of confounding was the use of NICU and hospital discharge as sample collection time points, rather than fixed postnatal ages, resulting in non-standardized timing across groups, particularly given the shorter length of stay in the KMC group. This may have introduced age-related confounding and should be considered when interpreting results. A further issue was the lack of detailed quantification of breastfeeding practices. Although human milk was provided to the entire cohort, the KMC group achieved a higher rate of exclusivity at discharge. Since breastfeeding is a known epigenetic modulator, it may mediate the effects of KMC. Without precise data on the breastfeeding ‘dose’ over time, we could not distinguish its independent effect from the overall effect of KMC.

Another relevant limitation was that ethical constraints prevented a randomized design; parents in the non-KMC group may have faced different socioeconomic or psychological stressors that also influence epigenetics. In addition, the inclusion of eight twin infants (four pairs), all in the KMC group, is a limitation, as observations within families are correlated. While our linear mixed-effects models included a random intercept per individual, they did not account for family-level clustering. However, since the study’s primary results were non-significant, it is unlikely that accounting for this clustering would have altered the overall conclusions, as adjusting for correlated data typically increases *p*-values. Nonetheless, this source of bias should be considered in future studies. Finally, we did not evaluate the *SLC6A4* promoter polymorphism, which is known to interact with both environmental stress and methylation levels.

Despite the limitations highlighted, this exploratory study contributes novel data to the understanding of the interaction between protective stress strategies and epigenetic programming in the neonatal period, supporting the identification of biomarkers and therapeutic targets to ensure the healthy neurodevelopment of preterm infants.

## 5. Conclusions

In conclusion, this longitudinal study did not find significant changes in *SLC6A4* methylation during the hospital stay of preterm infants, nor did it find robust differences between those exposed to or those not exposed to Kangaroo Mother Care. The overall stability of these epigenetic marks suggests that the serotonergic system’s promoter region in peripheral blood does not undergo substantial shifts during the neonatal period in response to KMC. However, these results should be interpreted with caution. They may be due to a genuine lack of effect, a follow-up period that was too short to detect epigenetic changes, or insufficient statistical power to detect subtle effects. On the other hand, the nominal difference in CpG6 methylation at hospital discharge and in CpG13 methylation trajectories—though isolated and exploratory—warrants further investigation. These findings suggest a possible targeted influence of KMC on *SLC6A4* methylation and may represent an early signal of the potential regulatory effects of nurturing care on the serotonergic system. However, the statistical strength of these associations is exploratory, and we frame our results as a foundational step toward future large-scale epigenetic trials in neonatal care. Replication in larger, multicenter samples with long-term follow-up periods and functional outcome measures is necessary to determine whether these subtle molecular signals are transient, biologically meaningful, or predictive of neurodevelopmental health in children born prematurely.

## Figures and Tables

**Table 1:** Maternal, gestational, and neonatal characteristics at birth in the Kangaroo Mother Care (KMC) and Non-KMC groups.

Characteristics	Total *n* = 75	KMC *n* = 51	Non-KMC *n* = 24	*p*-Value *
Maternal variables				
Maternal age, mean (SD)	26.4 (7.1)	26.3 (7.3)	26.8 (6.8)	0.787
Race/ethnicity, *n* (%)				
White	22 (29.3)	18 (35.3)	4 (16.7)	
Black	6 (8.0)	4 (7.8)	2 (8.3)	0.268
Brown-skinned (mixed-race ancestry)	47 (62.7)	29 (56.9)	18 (75.0)	
Level of Education, *n* (%)				
Higher Education (College)	3 (4.0)	3 (5.9)	0	
High School (complete)	33 (44.0)	25 (49.0)	8 (33.3)	0.015
Elementary School (complete)	39 (52.0)	23 (45.1)	16 (66.6)	
Marital status, *n* (%)				
Married	8 (10.7)	5 (9.8)	3 (12.5)	
Stabel union	40 (53.3)	34 (66.7)	6 (25.0)	0.002
Single	25 (33.3)	11 (21.6)	14 (58.3)	
Divorced	2 (2.7)	1 (2.0)	1 (4.2)	
Multiparous, *n* (%)	44 (58.7)	26 (51.0)	18 (75.0)	0.086
Underlying diseases ^a^, *n* (%)	14 (18.7)	9 (17.6)	5 (20.8)	0.758
Adverse conditions ^b^, *n* (%)	8 (10.7)	3 (5.9)	5 (20.8)	0.102
Prenatal consultations ≥ 6 (*n*%)	67 (89.3)	49 (96.1)	18 (75.0)	0.011
Prenatal corticosteroid, *n* (%)	60 (80.0)	42 (82.4)	18 (75.0)	0.540
Type of birth, *n* (%)				
Vaginal	34 (45.3)	23 (45.1)	11 (45.8)	1
Cesarean section	41 (54.7)	28 (54.9)	13 (54.2)	
Neonatal characteristics				
Sex, *n* (%)				
Male	34 (45.3)	21 (41.2)	13 (54.2)	0.421
Female	41 (54.7)	30 (58.8)	11 (45.8)	
Twins, *n* (%)	8 (10.7)	8 (15.7)	0	0.049
Birth weight (BW), median [IQR]	1.420 (1.238, 1.640)	1.440 (1.250, 1.665)	1.360 (1.15, 1.456)	0.092
Gestational age (GA), median [IQR]	31 (29, 32)	31 (29, 32)	30 (28, 32)	0.169
BW vs. GA, *n* (%)				
Adequate for Gestational Age	56 (74.7)	37 (72.5)	19 (79.2)	0.741
Small for Gestational Age	19 (25.3)	14 (27.5)	5 (20.8)	
Resuscitation in the delivery room, *n* (%)	25 (33.3)	18 (35.3)	7 (29.2)	0.793
Apgar score no 5’, median [min., max.]	9 (4, 10)	9 (4, 10)	9 (7, 9)	0.903

SD—Standard deviation; IQR—interquartile rate; ^a^ Underlying diseases (chronic hypertension, hypothyroidism, psychiatric disorders, diabetes, asthma, obesity); ^b^ Adverse conditions (childhood violence, domestic violence, illicit drugs, smoking, alcoholism, homelessness); * *p*-values (chi-square/Fisher’s exact test [categorical variables] and *t*-test/Mann–Whitney test [continuous variables]).

**Table 2:** Neonatal morbidities and outcomes in the Kangaroo Mother Care (KMC) and non-KMC groups.

Characteristics	Total (*n* = 75)	KMC (*n* = 51)	Non-KMC (*n* = 24)	*p*-Value *
CRIB, median (IQR)	1.0 (0.0, 3.0)	2.0 (0.0, 3.0)	1.0 (0.0, 3.0)	0.963
SNAPPE II, median (IQR)	20.0 (8.0, 24.0)	20.0 (8.0, 25.5)	15.0 (11.0, 23.0)	0.718
Higher NTISS, median (IQR)	12.0 (10.0, 15.0)	12.0 (10.0, 15.0)	13.5 (9.8, 16.0)	0.297
Early neonatal complications, *n* (%)	46 (61.3)	30 (58.8)	16 (66.7)	0.692
Respiratory distress syndrome	25 (33.3)	15 (29.4)	10 (41.7)	0.431
Pulmonary hemorrhage	3 (4.0)	3 (5.9)	0	0.054
Early sepsis	32 (42.7)	21 (42.1)	11 (45.8)	0.897
Patent ductus arteriosus	17 (22.7)	9 (17.6)	8 (33.3)	0.223
Shock	3 (4.0)	2 (3.9)	1 (4.2)	1
Late neonatal complications, *n* (%)	38 (50.7)	23 (45.1)	15 (62.5)	0.247
ICH/periventricular leukomalacia	31 (41.3)	18 (35.3)	13 (54.2)	0.195
Late sepsis	13 (17.3)	7 (13.7)	6 (25.0)	0.327
Bronchopulmonary dysplasia	10 (13.3)	4 (7.8)	6 (25.0)	0.066
Necrotizing enterocolitis	3 (4.0)	1 (2.0)	2 (8.3)	0.238
Retinopathy of prematurity	28 (37.3)	16 (31.4)	12 (50.0)	0.194
Treatment				
IMV, *n* (%)	27 (36.0)	16 (31.4)	11 (45.8)	0.338
IMV (days), median (IQR)	5.0 (2.0–14.0)	4.0 (1.8–11.3)	9.0 (4.0–6.5)	0.150
Antibiotics, *n* (%)	36 (48.0)	23 (45.1)	13 (54.2)	0.560
Systemic corticosteroid, *n* (%)	8 (10.7)	3 (5.9)	5 (20.8)	0.102
Parenteral nutrition, *n* (%)	49 (65.3)	29 (56.0)	20 (83.3)	0.047
Use of an umbilical venous catheter, *n* (%)	49 (65.3)	33 (64.7)	16 (66.7)	1
Use of an umbilical arterial catheter, *n* (%)	6 (8.0)	2 (3.9)	4 (16.7)	0.079
Use of PICC, *n* (%)	35 (46.7)	20 (39.2)	15 (62.5)	0.102
Red blood cell transfusion, *n* (%)	19 (25.3)	10 (19.6)	9 (37.5)	0.168
Hospital outcome, *n* (%)				
Hospital discharge	75 (100%)	51 (100%)	24 (100%)	-
NICU length of stay				
Days, median (IQR)	11.0 (7.0–26.0)	9.0 (6.0–18.0)	13.5 (9.5–39.5)	<0.01
Hospital length of stay				
Days, median (IQR)	38.0 (23.0–55.0)	29 (22.0–50.0)	43 (34.5–78.0)	<0.01

CRIB—Clinical Risk Index for Babies; SNAPPE—Score for Neonatal Acute Physiology-Perinatal Extension; NTISS—Neonatal Therapeutic Intervention Scoring System; IQR—interquartile rate; ICH—Intracranial hemorrhage; IMV—invasive mechanical ventilation; PICC—Peripherally Inserted Central Catheter. * *p*-values (chi-square/Fisher’s exact test [categorical variables] and *t*-test/Mann–Whitney test [continuous variables]).

**Table 3:** Duration and daily hours of in-hospital stages of the Kangaroo Mother Care stages.

	MEAN (SD)	MEDIAN (IQR)
Start day	5.0 (5.7)	3.0 (2.0–5.5)
Stage 1		
Duration in days	19.2 (16.8)	12.0 (8.5–21.5)
Average daily hours	2.3 (1.3)	2.0 (1.5–3.0)
Stage 2		
Duration in days	15.4 (7.2)	15.0 (9.0–20.0)
Average daily hours	5.3 (1.5)	6.0 (4.0–6.0)
Total in-hospital KMC		
Duration in days	34.7 (20.1)	29 (20.0–41.5)

**Table 4:** Comparison of the medians of total mean methylation of the *SLC6A4* gene between children in the Kangaroo Mother Care (KMC) and Non-KMC groups at different time points.

Characteristics	Total Cohort (%), Median (IQR)	KMC (%), Median (IQR)	Non-KMC (%), Median (IQR)	*p*-Value *
Day of birth	3.18 (2.39–3.54)	3.19 (2.36–3.59)	3.01 (2.53–3.50)	0.649
KMC (*n* = 51)				
Non-KMC (*n* = 23)				
NICU discharge	3.01 (2.64–3.39)	3.06 (2.65–3.47)	2.87 (2.62–3.17)	0.182
KMC (*n* = 50)				
Non-KMC (*n* = 23)				
Hospital discharge	3.01 (2.52–3.48)	3.01 (2.55–3.48)	3.13 (2.44–3.51)	0.955
KMC (*n* = 48)				
Non-KMC (*n* = 22)				

IQR—interquartile rate; NICU: Neonatal Intensive Care Unit; * Mann–Whitney U Test.

**Table 5:** Linear mixed-effects model estimates for *SLC6A4* methylation trajectories in KMC and non-KMC groups.

CpG	KMC Slope			Non-KMC Slope			Difference Between Slopes			Effect Size
	*ß*	95% CI	*p*	*ß*	95% CI	*p*	*ß*	95% CI	*p*	Adjusted Cohen d *
1	0.05066	−0.1810–0.2823	0.666	0.06895	−0.2759–0.4138	0.693	−0.01828	−0.4337–0.3971	0.397	−0.016
2	−0.05240	−0.3263–0.2215	0.706	0.04319	−0.3645–0.4509	0.834	−0.09558	−0.5867–0.3955	0.701	−0.070
3	0.01900	−0.2211–0.2591	0.876	−0.05390	−0.4107–0.3029	0.766	0.07289	−0.3571–0.5029	0.738	0.060
4	0.03117	−0.2001–0.2624	0.790	−0.05027	−0.3943–0.2937	0.773	0.08144	−0.3330–0.4959	0.698	0.070
5	−0.09349	−0.2493–0.0624	0.238	−0.07470	−0.3068–0.1575	0.526	−0.01879	−0.2984–0.2608	0.895	−0.024
6	−0.02734	−0.1690–0.1143	0.703	−0.05253	−0.2631–0.1581	0.623	0.02519	−0.2286–0.2790	0.845	0.035
7	−0.00134	−0.1442–0.1415	0.985	−0.01043	−0.2232–0.2023	0.923	0.00910	−0.2471–0.2653	0.944	0.013
8	−0.03298	−0.1535–0.0876	0.589	0.01507	−0.1643–0.1944	0.868	−0.04806	−0.2641–0.1680	0.661	−0.079
9	0.09140	−0.0915–0.2743	0.325	0.02521	−0.2471–0.2975	0.855	0.06620	−0.2618–0,3942	0.691	0.072
10	−0.08946	−0.2016–0.02271	0.117	0.01604	−0.1507–0.1828	0.850	−0.1055	−0.3064–0.0955	0.301	−0.187
11	0.04515	−0.0986–0.1889	0.536	0.03952	−0.1740–0.2531	0.715	0.00563	−0.2518–0.2630	0.966	0.008
12	−0.03273	−0.3939–0.3284	0.857	−0.06027	−0.5962–0.4757	0.823	0.02754	−0.6194–0.6745	0.933	0.022
13	−0.51600	−1.1471–0.1158	0.108	0.14870	−0.7871–1.0845	0.752	−0.66470	−1.7950–0.4655	0.245	0.344
Total	−0.04897	−0.1567–0.0588	0.3706	0.00557	−0.1550–0.1664	0.9444	−0.05465	−0.2482–0.1389	0.578	−0.101

*ß*—beta coefficient (slope estimates); 95% CI—95% confidence interval; KMC—Kangaroo Mother Care; *p*—*p*-value. Notes: Since no *p*-value was significant, no correction for multiple testing was performed. * Adjusted Cohen d—standardized effect size.

## Data Availability

The data presented in this study are available on request from the corresponding author due to privacy and ethical reasons.

## Supplementary Materials

The following supporting information can be downloaded at https://www.mdpi.com/article/10.3390/biomedicines14061269/s1, Table S1: Primers used for PCR and pyrosequencing. Table S2: Linear regressions for the association between methylation at NICU discharge and hospital discharge and KMC length of stay. Table S3: Duration of the in-hospital stages of the Kangaroo Mother Care (KMC). Table S4: *SLC6A4* promoter methylation in the KMC and Non-KMC groups at three time points. Table S5: Mixed effects models estimate of methylation change over time in the KMC and non-KMC group. Table S6: Mixed-effect models estimate comparing methylation changes over time between groups (reference: non-KMC group).

## Abbreviations

The following abbreviations are used in this manuscript:

5-HTP	5-hydroxytryptophan
5-HT	Serotonin
5-HTT	Serotonin transporter
BW	Birth weight
CRIB	Clinical Risk Index for Babies
CpG	Cytosine-phosphate-Guanine dinucleotide
DAG	Directed Acyclic Graph
D1	Day of birth
D2	Day of discharge from the Neonatal ICU
D3	Day of hospital discharge
DNA	Deoxyribonucleic acid
EDTA	Ethylenediaminetetraacetic Acid
FDR	False Discovery Rate
GA	Gestational age
gDNA	Genomic DNA
HPA	Hypothalamic–Pituitary–Adrenal (axis)
ICH	Intracranial hemorrhage
IMV	Invasive mechanical ventilation
IQR	Interquartile Range
KMC	Kangaroo Mother Care method
LMP	Last Menstrual Period
MAO	Monoamine Oxidase
NICU	Neonatal Intensive Care Unit
NTISS	Neonatal Therapeutic Intervention Scoring System
PICC	Peripherally Inserted Central Catheter
PCR	Polymerase Chain Reaction
REDCap	Research Electronic Data Capture
SAS	Statistical Analysis System
SD	Standard Deviation
*SLC6A4*	Solute Carrier Family 6 Member 4 (Serotonin Transporter Gene)
SNAPPE	Score for Neonatal Acute Physiology-Perinatal Extension
USG	Ultrasound
