# Supplementary material for: SLC6A4 Gene Methylation in Premature Infants Undergoing Kangaroo Mother Care: A Prospective Longitudinal Study
Source: Biomedicines. 2026 Jun 2;14(6):1269. doi: 10.3390/biomedicines14061269 (PMC13296614; doi:10.3390/biomedicines14061269)
Supplement: Supplementary file 1 [file biomedicines-14-01269-s001.zip › biomedicines-4296653-supplementary.pdf]

Supplementary material

Table S1. Primers used for PCR and pyrosequencing.

Primer	Sequence (5'-3')[a]	Sequence to analyze[b]	CpG sites	Product size
Forward	GGTTTTTATATGGTTTGATTTTTAGA	TYGTYGTTAA AGAGTTTTTG AAGAATTTTT G	1-2	70 bp
Reverse	/5Biosg/CAAAATAACCCAAAAATTCTTCAAAAACT			
Sequencing	TTTATATGGTTTGATTTTTAGATAG			
Forward	ATATGGTTTGATTTTTAGATAGTAGT	TTTTGYGTTA TTTTGAGGYG AATAAATTTA ATGTTTTTT YGYGGTYGYG GTTTYGYGTT TTYGTT	3-11	128 bp
Reverse	/5Biosg/AACCCAACCCCATCCAAC			
Sequencing	GTTAAAGAGTTTTTGAAGAAT			
Forward	TGAGGCG4AATAAATTTAATGTT	TTGYGTTYGT TAGGGAGGGG TYGYGTTAYG GGGYGGGGTG YGYGTTYGAT TTTAGA	12-13	132 bp
Reverse	/5Biosg/CCCCTCCTAACTCTAAAAT			
Sequencing	GTTTTAGTTGGATGGGG			

[a]Primers designed to target the first 13 CpG sites of the region 28562750-28562958 of chromosome 11 (GRCh37).
[b]CpG sites analyzed displayed as **Y**.

Table S2 – Linear regressions for the association between methylation at NICU discharge and hospital discharge and KMC length of stay.

	**Mehtylation at NICU discharge**			**Mehtylation at hospital discharge**		
	**Estimate**	**CI 95%**	***p-value***	**Estimate**	**CI 95%**	***p-value***
**CpG1**	-0.00	-0.03 – 0.02	*0.737*	0.00	-0.01 – 0.02	*0.509*
**CpG2**	-0.00	-0.03 – 0.03	*0.792*	-0.00	-0.02 – 0.02	*0.986*
**CpG3**	0.00	-0.03 – 0.03	*0.940*	0.00	-0.01 – 0.01	*0.843*
**CpG4**	0.01	-0.02 – 0.03	*0.731*	0.00	-0.01 – 0.01	*0.842*
**CpG5**	0.00	-0.01 – 0.01	*0.966*	-0.00	-0.02 – 0.01	*0.670*
**CpG6**	0.00	-0.01 – 0.01	*0.966*	-0.00	-0.02 – 0.01	*0.670*
**CpG7**	0.00	-0.01 – 0.02	*0.564*	0.00	-0.01 – 0.02	*0.767*
**CpG8**	0.00	-0.01 – 0.01	*0.714*	-0.00	-0.01 – 0.00	*0.323*
**CpG9**	0.01	-0.01 – 0.03	*0.447*	-0.00	-0.02 – 0.01	*0.654*
**CpG10**	0.00	-0.01 – 0.01	*0.572*	-0.00	-0.01 – 0.01	*0.676*
**CpG11**	-0.00	-0.01 – 0.01	*0.588*	0.01	-0.00 – 0.02	*0.159*
**CpG12**	0.01	-0.03 – 0.05	*0.725*	0.02	-0.03 – 0.07	*0.371*
**CpG13**	-0.02	-0.10 – 0.05	*0.549*	-0.03	-0.10 – 0.04	*0.379*
**Total Met**	-0.00	-0.01 – 0.01	0.945	-0.00	-0.01 – 0.01	*0.993*

Adjustment variables: Maternal (level of education, underlying diseases), gestational (prenatal visits) and neonatal (CRIB, sex, gestational age)

Table S3 – Duration of the in-hospital stages of the Kangaroo Mother Care (KMC)

Kangaroo Mother Care		
	**Mean (SD)**	**Median (IQR)**
**Start day**	5 (5,7)	3 (2; 5,5)
**Stage 1**		
Duration in days	19,2 (16,8)	12 (8,5; 21,5)
Average daily hours	2,3 (1,3)	2 (1,5; 3)
**Stage 2**		
Duration in days	15,4 (7,2)	15 (9; 20)
Average daily hours	5,3 (1,5)	6 (4; 6)
**Total in-hospital KMC**		
Duration in days	34,7 (20,1)	29 (20; 41,5)

Table S4 – *SLC6A4* promoter methylation in the KMC and Non-KMC groups at three time points.

		Total cohort (%) median (IQR)	KMC (%) median (IQR)	Non-KMC (%) median (IQR)	*p-value*[a]
**Day of birth** KMC (n=51) Non-KMC (n=23)	CpG 1	2.14 (1.52-2.76)	2.22 (1.65-2.70)	1.80 (1.35-2.88)	*0.834*
	CpG 2	2.28 (1.62-2.96)	2.25 (1.69-2.99)	2.37 (1.56-2.89)	*0.958*
	CpG 3	2.10 (1.64-2.57)	2.02 (1.59-2.63)	2.20 (1.91-2.38)	*0.834*
	CpG 4	2.18 (1.73-2.72)	1.93 (1.55-2.62)	2.21 (2.02-2.89)	*0.611*
	CpG 5	2.44 (2.07-3.22)	2.40 (2.09-3.01)	2.56 (1.95-3.55)	*0.834*
	CpG 6	1.12 (0.89-1.32)	1.09 (0.86-1.23)	1.25 (0.94-1.94)	*0.611*
	CpG 7	1.27 (1.07-1.82)	1.26 (1.10-1.84)	1.31 (1.03-1.68)	*0.958*
	CpG 8	0.87 (0.59-1.23)	0.88 (0.59-1.24)	0.86 (0.63-1.21)	*0.958*
	CpG 9	2.18 (1.75-2.67)	2.12 (1.59-2.49)	2.33 (1.97-3.10)	*0.611*
	CpG 10	0.98 (0.70-1.32)	0.94 (0.62-1.33)	1.01 (0.80-1.23)	*0.834*
	CpG 11	1.52 (1.21-1.90)	1.52 (1.21-2.03)	1.53 (1.27-1.79)	*0.958*
	CpG 12	8.15 (6.89-10.61)	8.06 (6.80-11.06)	8.24 (7.25-9.65)	*0.958*
	CpG 13	9.66 (4.39-14.41)	10.76 (4.58-14.78)	7.50 (4.63-12.21)	*0.834*
	Total Met	3.18 (2.39 - 3.54)	3.19 (2.36 - 3.59)	3.01 (2.53 - 3.50)	*0.649*
**NICU discharge** KMC (n=50) Non-KMC (n=23)	CpG1	2.22 (1.65-2.70)	2.17 (1.77-2.98)	2.08 (1.90-2.53)	*0.957*
	CpG 2	2.35 (1.79-3.08)	2.35 (1.75-3.17)	2.35 (1.82-2.91)	*0.957*
	CpG 3	2.22 (1.77-2.91)	2.21 (1.73-3.03)	2.29 (1.88-2.58)	*0.957*
	CpG 4	2.05 (1.81-2.55)	2.02 (1.81-2.34)	2.11 (1.82-2.65)	*0.957*
	CpG 5	2.44 (2.07-2.88)	2.44 (2.07-2.93)	2.31 (2.08-2.65)	*0.957*
	CpG 6	0.96 (0.78-1.32)	0.96 (0.74-1.40)	1.04 (0.80-1.25)	*0.957*
	CpG 7	1.39 (1.08-1.84)	1.41 (1.09-1.75)	1.38 (1.10-1.88)	*0.957*
	CpG 8	0.87 (0.62-1.20)	0.92 (0.62-1.22)	0.82 (0.64-1.17)	*0.957*
	CpG 9	2.17 (1.79-2.91)	2.17 (1.81-3.12)	2.15 (1.71-2.87)	*0.957*
	CpG 10	0.91 (0.70-1.22)	0.90 (0.69-1.27)	1.01 (0.75-1.22)	*0.957*
	CpG 11	1.48 (1.22-1.79)	1.48 (1.20-1.78)	1.49 (1.23-1.87)	*0.957*
	CpG 12	7.82 (7.19-10.40)	7.86 (7.27-10.35)	7.58 (6.99-9.55)	*0.957*
	CpG 13	8.99 (5.53-12.86)	10.02 (4.54-13.40)	7.49 (4.52-11.71)	*0.957*
	Total Met	3.01 (2.64 - 3.39)	3.06 (2.65 - 3.47)	2.87 (2.62 - 3.17)	*0.182*
**Hospital discharge** KMC (n=48) Non-KMC (n=22)	CpG 1	2.37 (1.89-2.94)	2.41 (2.04-2.97)	2.09 (1.66-2.70)	*0.647*
	CpG 2	2.42 (1.86-2.99)	2.24 (1.80-3.06)	2.56 (2.02-2.89)	*0.846*
	CpG 3	2.29 (1.85-2.82)	2.29 (1.92-2.83)	2.18 (1.51-2.80)	*0.647*
	CpG 4	2.30 (1.88-2.72)	2.36 (1.86-2.73)	2.21 (2.10-2.68)	*0.934*
	CpG 5	2.58 (2.32-2.95)	2.52 (2.33-2.84)	2.78 (2.27-3.01)	*0.846*
	CpG 6	1.04 (0.86-1.34)	0.96 (0.82-1.26)	1.21 (1.02-1.41)	*0.273*
	CpG 7	1.24 (1.03-1.80)	1.24 (0.99-1.75)	1.24 (1.08-1.90)	*0.846*
	CpG 8	0.82 (0.64-1.10)	0.79 (0.66-1.09)	0.89 (0.60-1.11)	*0.846*
	CpG 9	2.34 (1.97-2.76)	2.28 (1.97-2.71)	2.52 (1.93-2.92)	*0.846*
	CpG 10	0.88 (0.67-1.15)	0.78 (0.67-1.11)	0.98 (0.80-1.23)	*0.533*
	CpG 11	1.49 (1.33-1.83)	1.47 (1.30-1.85)	1.55 (1.36-1.82)	*0.846*
	CpG 12	7.84 (6.81-10.69)	8.02 (7.14-10.50)	7.19 (6.73-10.67)	*0.846*
	CpG 13	7.89 (4.73-12.70)	8.49 (4.91-12.63)	7.36 (4.33-12.70)	*0.846*
	Total Met	3.01 (2.52 - 3.48)	3.01 (2.55 - 3.48)	3.13 (2.44 - 3.51)	*0.955*

IQR – Interquartile range; NICU: Neonatal Intensive Care Unit

[a]Mann-Whitney U test. p-values adjusted by the Benjamin-Hochberg method (False Discovery Rate – FDR).

Table S5. Mixed effects models estimate of methylation change over time in the KMC and non-KMC group.

CpG	Day	KMC group				Non-KMC group			
		Crude mean (SD)	beta	SE	*p-value**	Crude mean (SD)	beta	SE	*p-value**
CpG1	D1	2.49 (1.64)				2.09 (0.89)			
	D2	2.71 (1.88)	0.0532	0.1345	*0.9884*	2.21 (0.82)	0.0676	0.0919	*0.9081*
	D3	2.57 (1.06)				2.22 (0.73)			
CpG2	D1	2.73 (1.84)				2.38 (0.96)			
	D2	2.94 (2.24)	-0.0471	0.1603	*0.9884*	2.43 (0.76)	0.0389	0.0988	*0.9081*
	D3	2.59 (1.30				2.47 (0.87)			
CpG3	D1	2.28 (1.28)				2.24 (0.61)			
	D2	2.72 (2.19)	0.0157	0.0142	*0.9884*	2.33 (0.54)	-0.0469	0.0743	*0.9081*
	D3	2.34 (0.66)				2.13 (0.79)			
CpG4	D1	2.29 (1.26)				2.45 (0.73)			
	D2	2.34 (2.19)	0.0312	0.1368	*0.9884*	2.31 (0.67)	-0.0558	0.0733	*0.9081*
	D3	2.37 (0.66)				2.37 (0.70)			
CpG5	D1	2.76 (1.09)				2.81 (0.89)			
	D2	2.79 (1.34)	-0.0948	0.0883	*0.9884*	2.42 (0.53)	-0.0750	0.0767	*0.9081*
	D3	2.59 (0.63)				2.66 (0.52)			
CpG6	D1	1.14 (0.67)				1.49 (0.84)			
	D2	1.16 (0.70)	-0.0255	0.0741	*0.9884*	0.98 (0.39)	-0.0547	0.0987	*0.9081*
	D3	1.09 (0.98)				1.40 (0.81)			
CpG7	D1	1.44 (0.81)				1.41 (0.73)			
	D2	1.60 (1.01)	-0.0005	0.0776	*0.9944*	1.47 (0.67)	-0.0076	0.0873	*0.9310*
	D3	1.44 (0.96)				1.41 (0.58)			
CpG8	D1	0.96 (0.78)				0.87 (0.41)			
	D2	1.04 (0.80)	-0.0313	0.0656	*0.9884*	0.89 (0.54)	0.0155	0.0733	*0.9081*
	D3	0.89 (0.63)				0.91 (0.56)			
CpG9	D1	2.28 (1.08)				2.61 (1.03)			
	D2	2.61 (1.38)	0.0927	0.1025	*0.9884*	2.23 (0.81)	0.0245	0.0967	*0.9081*
	D3	2.47 (0.99)				2.67 (1.04)			
CpG10	D1	1.02 (0.66)				1.11 (0.63)			
	D2	1.04 (0.76)	0.0900	0.0600	*0.8873*	0.95 (0.38)	0.0150	0.0730	*0.9081*
	D3	0.83 (0.44)				1.14 (0.62)			
CpG11	D1	1.63 (0.70)				1.52 (0.67)			
	D2	1.66 (0.89)	0.0437	0.0742	*0.9884*	1.69 (0.84)	0.0366	0.0989	*0.9081*
	D3	1.73 (0.85)				1.60 (0.37)			
CpG12	D1	9.31 (3.23)				8.88 (2.46)			
	D2	9.10 (2.86)	-0.0267	0.1746	*0.9884*	8.60 (2.67)	-0.0567	0.1592	*0.9081*
	D3	9.23 (3.09)				8.81 (3.24)			
CpG13	D1	10.55 (5.83)				9.06 (4.93)			
	D2	9.49 (5.02)	-0.5227	0.3149	*0.8873*	9.12 (5.78)	0.1397	0.4595	*0.9081*
	D3	9.25 (4.67)				9.55 (6.30)			
Total mean met.	D1	3.14 (0.88)				2.99 (0.57)			
	D2	3.18 (0.82)	-0.0485	0.0620	*0.4261*	2.89 (0.52)	0.0027	0.0458	*0.9535*
	D3	3.03 (0.60)				3.03 (0.67)			

NICU – Neonatal intensive care unit; NTISS – Neonatal Therapeutic Intervention Scoring System; KMC – Kangaroo Mather Care; SD – Standard Deviation; SE – Standard Error.
Adjustment variables: sex, gestational age, worst NTISS, early and late neonatal complications.
*p-values adjusted by the Benjamin-Hochberg method (False Discovery Rate – FDR).

Table S6. Mixed-effect models estimate comparing methylation changes over time between groups (reference: non-KMC group)

CpG	Day	KMC group		Non-KMC group		KMC compared to non-KMC		
		Crude mean (SD)	△	Crude mean (SD)	△	beta	SE	*p-value**
CpG1	D1	2.49 (1.64)	-	2.09 (0.89)	-	-0.0183	0.2101	*0.9656*
	D2	2.71 (1.88)	0.22	2.21 (0.82)	0.12			
	D3	2.57 (1.06)	0.08	2.22 (0.73)	0.13			
CpG2	D1	2.73 (1.84)	-	2.38 (0.96)	-	-0.09558	0.2484	*0.9656*
	D2	2.94 (2.24)	0.21	2.43 (0.76)	0.05			
	D3	2.59 (1.30)	-0.14	2.47 (0.87)	0.09			
CpG3	D1	2.28 (1.28)	-	2.24 (0.61)	-	0.07289	0.2175	*0.9656*
	D2	2.72 (2.19)	0.44	2.33 (0.54)	0.09			
	D3	2.34 (0.66)	0.06	2.13 (0.79)	-0.11			
CpG4	D1	2.29 (1.26)	-	2.45 (0.73)	-	0.08144	0.2096	*0.9656*
	D2	2.34 (2.19)	0.05	2.31 (0.67)	-0.14			
	D3	2.37 (0.66)	0.08	2.37 (0.70)	-0.08			
CpG5	D1	2.76 (1.09)	-	2.81 (0.89)	-	-0.01879	0.1414	*0.9656*
	D2	2.79 (1.34)	0.03	2.42 (0.53)	-0.39			
	D3	2.59 (0.63)	-0.17	2.66 (0.52)	-0.15			
CpG6	D1	1.14 (0.67)	-	1.49 (0.84)	-	0.02519	0.1284	*0.9656*
	D2	1.16 (0.70)	0.02	0.98 (0.39)	-0.51			
	D3	1.09 (0.98)	-0.05	1.40 (0.81)	-0.09			
CpG7	D1	1.44 (0.81)	-	1.41 (0.73)	-	0.009096	0.1296	*0.9656*
	D2	1.60 (1.01)	0.16	1.47 (0.67)	0.06			
	D3	1.44 (0.96)	0	1.41 (0.58)	0			
CpG8	D1	0.96 (0.78)	-	0.87 (0.41)	-	0.04806	0.1093	*0.9656*
	D2	1.04 (0.80)	0.08	0.89 (0.54)	0.02			
	D3	0.89 (0.63)	-0.07	0.91 (0.56)	0.04			
CpG9	D1	2.28 (1.08)	-	2.61 (1.03)	-	0.06620	0.1659	*0.9656*
	D2	2.61 (1.38)	0.33	2.23 (0.81)	-0.38			
	D3	2.47 (0.99)	0.19	2.67 (1.04)	0.06			
CpG10	D1	1.02 (0.66)	-	1.11 (0.63)	-	-0.1055	0.1016	*0.9656*
	D2	1.04 (0.76)	0.02	0.95 (0.38)	-0.16			
	D3	0.83 (0.44)	-0.19	1.14 (0.62)	0.03			
CpG11	D1	1.63 (0.70)	-	1.52 (0.67)	-	0.005630	0.1302	*0.9656*
	D2	1.66 (0.89)	0.03	1.69 (0.84)	0.17			
	D3	1.73 (0.85)	0.1	1.60 (0.37)	0.08			
CpG12	D1	9.31 (3.23)	-	8.88 (2.46)	-	0.02754	0.3241	*0.9656*
	D2	9.10 (2.86)	-0.21	8.60 (2.67)	-0.28			
	D3	9.23 (3.09)	-0.08	8.81 (3.24)	-0.07			
CpG13	D1	10.55 (5.83)	-	9.06 (4.93)	-	-0.6647	0.5663	*0.9656*
	D2	9.49 (5.02)	-1.06	9.12 (5.78)	0.06			
	D3	9.25 (4.67)	-1.3	9.55 (6.30)	0.49			
Total mean met.	D1	3.14 (0.88)	-	2.99 (0.57)	-	-0.05465	0.0979	*0.5775*
	D2	3.18 (0.82)	0.04	2.89 (0.52)	-0.10			
	D3	3.03 (0.60)	-0.11	3.03 (0.67)	0.04			

CC – Confidence interval; NICU – Neonatal intensive care unit; NTISS – Neonatal Therapeutic Intervention Scoring System; KMC – Kangaroo Mather Care; met. – Methylation; SD – Standard deviation; SE – Standard error

△ Percentage change in methylation over time

*p-values adjusted by the Benjamin-Hochberg method (False Discovery Rate – FDR)
